# Clocks in Dreams: Analysis of a Long Dream Series

**DOI:** 10.3390/clockssleep3040043

**Published:** 2021-11-24

**Authors:** Michael Schredl

**Affiliations:** Sleep Laboratory, Central Institute of Mental Health, Medical Faculty Mannheim, Heidelberg University, P.O. Box 12-21-20, 68072 Mannheim, Germany; Michael.Schredl@zi-mannheim.de; Tel./Fax: +49-621-1703-1782

**Keywords:** dream content, clocks, continuity hypothesis

## Abstract

Many dream content analytic studies focus on dream characters, animals, social interactions and so on, but they rarely analyze the frequency of everyday objects in dreams. In the present paper, the frequency and phenomenology of clock dreams in a dream series of 12,476 dreams of a single male dreamer was analyzed. The clock dreams (0.74% of all dreams) show a variety of contexts not only related to the time management of the dreamer within the dream. Interestingly, clocks that belong to the dreamer in waking life occurred very rarely in his dreams. Given that keeping time schedules and appointments in waking life is of importance to almost everyone, the low frequency of clock dreams might be explained by novelty, that is, waking-life experiences that repeat themselves regularly do not show up in dreams that often. Thus, studying everyday objects such as clocks in dreams might help refine the current models describing the continuity between waking and dreaming.

## 1. Introduction

Many biological organisms are governed by molecular clocks that are synchronized with the 24-h day–night rhythm [1]. In human society, external clocks are very important for timekeeping, e.g., school and working hours, train schedules, dentist appointments [2]. The history of measuring time with clocks dates far back in history, starting with sun dials and continuing with the high-precision atomic clocks used today [2]. Even in the area of mobile phones, many individuals use clocks, e.g., in the sample (*N* = 3084) of Montag et al. [3], 45% of the participants regularly wore a wristwatch and 67% had an alarm clock for waking up in the morning. According to the continuity hypothesis of dreaming [4,5], we dream about topics that are important to us, e.g., family members [6], spouses [7], sexuality [8], our own children [9], pets [10] and work [11]. Given that punctuality and timekeeping is important (see above), the question arises as to how often clocks for measuring time occur in dreams. Interestingly, this has never been studied systematically. In a sample of 1000 dreams reported by 100 male and 100 female students, clocks or watches (timepieces) were present in seven dreams, a percentage of 0.7% [6]. The author conducted a word search and manual evaluation of the occurrences within the dream series of “Barb Sanders”, available on dreambank.net (accessed on 20 July 2021) (see also: [12,13]), using “clock”, “watch” and “alarm”. Overall, clocks (e.g., grandfather clock) and/or watches (often “my watch”) were found in 45 of the 4254 dreams (1.06%).

The present analysis of a long dream series (*N* = 12,476 dreams) aimed at studying the frequency and phenomenology of clock dreams, e.g., does the clock/watch belong to the dreamer or does the clock play a role for the time management within the dream.

## 2. Method

### Participant and Dream Diary

The male participant started keeping an unstructured dream diary from the age of 22, with the first dream recorded on 5 September 1984. The dreamer rarely wore a wristwatch but used an alarm clock in the morning and a portable alarm clock to keep appointments. He did not possess a smart phone or a similar device displaying time. He has no history of mental disorders and his field of work is psychological research (not related to time perception or something similar). For the present analysis, all 12,476 dreams recorded between the first dream and 10 July 2016 (the current status of digitalized and coded dreams) were included. The mean length of the dreams was 137.27 ± 85.53 words.

## 3. Procedure

Dream reports were originally handwritten but were then typed and entered into a database (Alchera 3.72, created by Harry Bosma, www.mythwell.com, (last accessed on 24 November 2021) by the dreamer himself. This database permits the assigning of keywords to the dreams, a task that was also carried out by the dreamer. Each dream was coded by the dreamer while typing the dreams for the presence of clocks, watches, etc. In a second step, the dreamer classified these dreams according to several parameters: (1) type of clock/watch (watch, alarm clock, public clock, church clock, wall clock, other clock/watch, and unspecified), (2) is the clock relevant for time management within the dream, e.g., dreamer looks at a clock and realizes he has to hurry, (3) familiarity of the clock (clock/watch belongs to the dreamer, dreamer is familiar with the clock, e.g., belonging to another person, unfamiliar clock) and (4) bizarreness of the clock (clock/watch that is realistic, clock/watch is bizarre). If the clock/watch belongs to the dreamer within the dream, it was also noted whether or not it was a watch/clock the dreamer used in waking life.

The Alchera software provides a word count for each dream report. Reports included only dream experience-related words and all redundancies were excluded, e.g., repetitions that occurred in writing down the dream in the morning. The analysis unit was an individual dream report. The data were exported into an Excel spreadsheet (Microsoft) and the descriptive data analysis was carried out using the SAS 9.4 software package for Windows (Cary, NC, USA).

## 4. Results

Overall, 0.74% of the dreams included some type of clock/watch. The average word count of these 92 dreams was 191.77 ± 79.37 words. If the clock/watch in the dream was specified, watches and alarm clocks occurred most often (see Table 1). Public clocks, church clocks and wall clocks were seldom part of a dream. Examples for other watches/clocks were stopwatches, chess clocks and mechanical clocks. In 16 dreams, the clock/watch belonged to the dreamer; however, in only four occurrences was it a watch/clock that the dreamer possessed in waking life, e.g., golden wristwatch of his grandfather. In most cases, the dreamer was not familiar with the clock/watch in the dream. The clock/watch was not important for the time planning within every dream (see Table 1). In dream example 1, however, the clock did play a role for time management within the dream. On the other hand, his clock dreams also included other topics, such as buying a watch in a shop or returning a lost watch. Very rarely, the clock/watch within the dream showed bizarre features (see dream examples 3 and 4 below). Dream examples 5 and 6 include clocks within different contexts, uncertainty (dream example 5) and disturbance (dream example 6).

- Dream example 1: “It’s just after 9:30. I check the clock and my schedule. At 9:45 school starts. I get ready. When I look at the clock again, I notice that I made a mistake in the day; today I have already missed two hours. …”
- Dream example 2: “… Then I come to D.’s room. She has just got up and shows me a watch that is particularly close to her heart, a silver-colored Swiss wristwatch. …”
- Dream example 3: “… On the way to the building, I call Ruth on the phone integrated into my wristwatch. …” (Dream was recorded in 1995).
- Dream example 4: “… The man sees my little radio alarm clock that is on the table. My mother said she was really tired today. We conclude that the time change (daylight saving) last night is responsible and therefore an hour is missing. My radio alarm clock automatically updated the time. The display shows things that I have never seen before. I want to get it back to normal, which is not that easy. The man picks it up and quickly presses the keys so that everything is okay again. I ask him if you can get these menus with very short, quick presses. He says yes. I don’t want to dig deeper into the subject because I wouldn’t normally need these menus. It’s very kind of the man to help me. …”
- Dream example 5: “… I look at two clocks, but they show different times, one with something at 10 p.m. I want to find another clock, get up. … In the next room (belonging to my sister’s family) is a large computer … I also find a clock that shows the correct time.”
- Dream example 6: “I am in a large courtyard, in front of the window (large) of my ground floor apartment (old building). There is still something to do with people I know; I then want to go to the apartment. … Above the door to the bedroom there is a small, old clock whose clockwork (with a hand showing seconds) is quite annoying (noise). I wonder if that could bother me at night, since it is not within my bedroom, it could work. In the room, to the left of the front wall opposite the open window, there is a large, artistically crafted clockwork that runs silently. I’ll take a closer look. I haven’t lived in the apartment that long and don’t know what the noise level of the clock is like at night.”

## 5. Discussion

The findings based on a dream series indicate that clocks are present in 0.74% of the dreams. Despite clocks being an important everyday object, especially the clocks the dreamer owns and uses, these clocks rarely occur in dreams. The varieties of clocks and contexts of the clocks within the dream scenario are very large, indicating that clock dreams might not have simple meanings.

The frequency of the clock dreams in which it plays a role in the time management of the dreamer is relatively low (0.43%), just about 60% of all clock dreams. Even though the dreamer owned a watch in twelve dreams, only in four dreams of the total dream series is the watch within the dream actually a watch the dreamer owns in real life. Despite the importance of clocks in keeping appointments and schedules [2], this everyday object occurs very rarely in dreams. Glasses, for example, also an everyday object that is important for this shortsighted dreamer, were found in about 1% of the dreams. As the mathematical model of the continuity hypothesis postulated that the emotional intensity of the waking-life experience increases the chance of it being incorporated into subsequent dreams [5], one might assume that time management, overall, is not that important to people. However, about 24% report recurrent nightmares of being late, one of the top five nightmare themes [14]—suggesting that keeping appointments and punctuality are important, especially if something is going wrong—such as being late. In view of this finding, the explanation that clocks are rare in dreams because of low salience in waking life might not be that plausible.

Another factor that might offer an explanation is novelty. Managing time (in most times, successfully) is something that everyone has to undertake every day for long time periods, e.g., getting up in the morning for attending school—often aided by an alarm clock. Therefore, an individual is normally used to keeping time schedules and using his or her watch/clock and these repetitive experiences might not show up in dreams very often. These theoretical considerations clearly indicate that further studies of everyday objects in dreams—although systematic research in this area is very scarce—can be very stimulating for theories modeling the relationship between waking life and dreaming by identifying factors, such as novelty or emotional salience, that affect the continuity between waking and dreaming.

Interestingly, the clocks within the dreams were very rarely bizarre. This is in contrast with elevators analyzed within in the same dream series [15]; about 43% of the elevators in dreams had bizarre features (moving in unexpected directions, even flying, for example) often associated with inducing anxiety within the dreamer. As dreams have been described as creative [16,17], it would be very interesting to study why some everyday objects show up in dreams exactly as they are in waking life (most often as a generic category, not as a specific and familiar object from waking life). One line of thinking might be that the bizarreness is not a feature of the object itself but related to the usage of the object, e.g., elevators move in a bizarre way but might not be bizarre regarding their physical properties.

There is a long history of linking dream elements to waking life, e.g., the dream books of Artemidorus of Daldis [18]. In a modern version of a dream symbol dictionary, the following “meaning” of clocks in dreams can be found:

“Clock. Deadlines, running out of time; an acute awareness of the passing of time. Clock dreams often occur when one is faced with serious and life-threatening illness, or is close to someone who is in such a situation. Trying to stop a clock, or a clock breaking, can represent a fear of death. Clocks gone out of control symbolize unresolved fears and anxieties. Another meaning: A woman’s biological clock, that is, her time remaining to conceive and bear children [19] (p. 183)”.

These interpretations focus mainly on the clock within the context of time management (e.g., the metaphorical expression “Your time is up” for death) but the dream examples above clearly indicate that clocks in dreams occur in a variety of contexts, e.g., confusion (different clocks showing different times), possible disturbance by clock noise, clock as something valuable or wristwatch as a phone. That is, the variety of clocks and their contexts within the dream clearly indicate that simple interpretations are not helpful. For example, several interpretations do not fit the present (male) dreamer who was quite healthy and was not close to any terminally ill persons.

## 6. Limitations

The main limitation of the study is the fact that all the dreams were provided by a single dreamer. Interestingly, the frequency of clock dreams in this series (0.74%) is roughly comparable with previous findings: 0.7% in a student sample [6] and 1.06% in another long dream series (see introduction). That is, regarding the frequency of clock dreams, the bias seems small.

The present analysis is not a medical case report for which specific guidelines have been developed [20]. The major aim was to address a topic, namely clocks in dreams, that has never been addressed before. That is, the paper describes an exploratory study presenting descriptive statistics. Second, the analysis relied on a dream journal kept by the dreamer for personal reasons, not explicitly for this specific study. On the one hand, this has disadvantages, as there is no standardization of dream reporting (e.g., recording times, sleep duration, life circumstances, etc.)—all factors that might have had an effect on dream content. On the other hand, the retrospective analysis also offers advantages. Studying the frequency of music dreams, for example, preplanned and with probing questions about possible contents related to music yielded much higher percentages of music dreams [21] compared to analyzing dream diaries kept for other reasons [22]: 20% vs. 8%. That is, directing the focus of the participants can actually bias the findings, see also [23]. Person-related variables, such as profession, might also have an effect on dream content in general and on clock dreams in particular; therefore, it would be very interesting to study a variety of persons, e.g., persons who wear a wristwatch regularly or professionals for whom keeping time schedules plays a crucial role, e.g., security guards or tram drivers. Dream diaries kept in the home setting typically elicit dreams from late REM periods as these are most easily remembered after spontaneous awakenings. Even though content differences between home and laboratory-collected dreams regarding general topics are not very pronounced (less aggressive and sexual interactions in lab dreams) [24], the problem is that dreams collected in the laboratory also often include reference to the experimental situation (electrodes, sleep lab, experimenter, etc.) [25]. Thus, laboratory dreams might also be biased regarding the occurrence of clocks in dreams as strict time-locked experimental protocols (bed times, awakenings times) are implemented in the sleep laboratory.

## 7. Conclusions

To summarize, everyday objects such as clocks do occur in dreams in a variety of contexts. Interestingly, the dreamer’s own watches/clocks occur very rarely in dreaming, indicating that novelty of the waking-life experience might be a factor affecting the continuity between waking and dreaming. Although such everyday objects such as clocks occur quite seldom in dreams when compared to family members (18.71% [26]), romantic partners (6.41% to 22.69% [27]) and former school mates (5.90% [28]) in the same dream series, studying the frequency and phenomenology of dreams incorporating such objects might help refine the theories modeling the relationship between waking life and dreaming. In addition, analyzing clock dreams might help obtain a better understanding of time perception or sense of time in dreams, a topic that has been studied in lucid dreams, showing that sense of time in lucid dreams is comparable to waking life [29].

## Figures and Tables

**Table 1:** Characteristics of the dreams with clocks/watches (*N* = 92).

Characteristics	*N*	Percent
**Type of Clock/Watch**		
Watch	20	21.7%
Alarm clock	17	18.5%
Public clock	7	7.6%
Church clock	4	4.4%
Wall clock	7	7.6%
Other clocks	13	14.1%
Unspecified	24	26.1%
**Familiarity**		
Clock/watch belongs to the dreamer	16	17.4%
Clock/watch is familiar but not belongs to the dreamer	5	5.4%
Unfamiliar clock	71	77.2%
**Bizarreness**		
Realistic clock/watch	88	95.7%
Bizarre clock/watch	4	4.4%
**Clock/Watch Is Relevant for Time Management within the Dream**		
Yes	54	58.7%
No	38	41.3%

## Data Availability

As the dream reports include personal data like names and places, the raw data are not available on request; solely the classifications that served as basis for the descriptive statistics can be requested.
